# Glucose transport engineering allows mimicking fed-batch performance in batch mode and selection of superior producer strains

**DOI:** 10.1186/s12934-022-01906-1

**Published:** 2022-09-07

**Authors:** Daniela Velazquez, Juan-Carlos Sigala, Luz María Martínez, Paul Gaytán, Guillermo Gosset, Alvaro R. Lara

**Affiliations:** 1 Departamento de Procesos y Tecnología, Universidad Autónoma Metropolitana, Vasco de Quiroga 4871, 05348 Mexico City, Mexico; 2 Departamento de Ingeniería Celular y Biocatálisis, Instituto de Biotecnología, Universidad Nacional Autónoma de México, Avenida Universidad 2001, Col. Chamilpa, 62210 Cuernavaca, MOR Mexico

**Keywords:** Overflow metabolism, Fed-batch, High cell-density, Glucose transport, Cell engineering

## Abstract

**Background:**

Fed-batch mode is the standard culture technology for industrial bioprocesses. Nevertheless, most of the early-stage cell and process development is carried out in batch cultures, which can bias the initial selection of expression systems. Cell engineering can provide an alternative to fed-batch cultures for high-throughput screening and host selection. We have previously reported a library of *Escherichia coli* strains with single and multiple deletions of genes involved in glucose transport. Compared to their wild type (W3110), the mutant strains displayed lower glucose uptake, growth and aerobic acetate production rates. Therefore, when cultured in batch mode, such mutants may perform similar to W3110 cultured in fed-batch mode. To test that hypothesis, we evaluated the constitutive expression of the green fluorescence protein (GFP) in batch cultures in microbioreactors using a semi defined medium supplemented with 10 or 20 g/L glucose + 0.4 g yeast extract/g glucose.

**Results:**

The mutant strains cultured in batch mode displayed a fast-growth phase (growth rate between 0.40 and 0.60 h^−1^) followed by a slow-growth phase (growth rate between 0.05 and 0.15 h^−1^), similar to typical fed-batch cultures. The phase of slow growth is most probably caused by depletion of key amino acids. Three mutants attained the highest GFP fluorescence. Particularly, a mutant named WHIC (Δ*ptsHIcrr*, Δ*mglABC*), reached a GFP fluorescence up to 14-fold greater than that of W3110. Strain WHIC was cultured in 2 L bioreactors in batch mode with 100 g/L glucose + 50 g/L yeast extract. These cultures were compared with exponentially fed-batch cultures of W3110 maintaining the same slow-growth of WHIC (0.05 h^−1^) and using the same total amount of glucose and yeast extract than in WHIC cultures. The WHIC strain produced approx. 450 mg/L GFP, while W3110 only 220 mg/L.

**Conclusion:**

The combination of cell engineering and high throughput screening allowed the selection of a particular mutant that mimics fed-batch behavior in batch cultures. Moreover, the amount of GFP produced by the strain WHIC was substantially higher than that of W3110 under both, batch and fed-batch schemes. Therefore, our results represent a valuable technology for accelerated bioprocess development.

**Supplementary Information:**

The online version contains supplementary material available at 10.1186/s12934-022-01906-1.

## Background

Production of recombinant proteins using *Escherichia coli* (*E. coli*) is a technology well established at industrial level. To attain high biomass yields and recombinant protein productivities, high cell-density cultures are used. These cultures require large amounts of glucose, which is the preferred carbon and energy source for this bacterium. In batch cultures, the glucose uptake rate (*q*_*S*_) reaches its maximum value, which in turn triggers the synthesis of acetate, even if oxygen is present in sufficient amounts, a phenomenon known as overflow metabolism [1, 2]. Acetate accumulation is undesired, because it negatively affects bioprocess performance. Therefore, controlling the *q*_*S*_ below a certain threshold value to avoid overflow metabolism while keeping the maximum growth rate is highly desirable. Maintaining the *q*_*S*_ below the threshold value is normally achieved by controlling the glucose supply in the so-called fed-batch scheme [3]. In the case of exponential feeding, the rate of nutrient addition increases in proportion to cell growth, in such a way that a certain specific growth rate (*µ*_*set*_ < *µ*_*max*_) can be maintained and the substrate concentration in the medium is close to zero [3]. Because maximum productivities and yields can be obtained at lower growth rates than those displayed in batch cultures [4, 5], exponentially fed-batch cultures can be operated to maintain the growth rate that maximizes productivity, while overflow metabolism is avoided, and the specific oxygen uptake rate is reduced. However, fed-batch cultures have certain disadvantages, such as the requirement of additional equipment and control systems, which hinders its implementation in early-stage process development, particularly if high throughput screening of cell factories and gene circuits are sought.

Due to the importance of fed-batch cultures, several approaches have been proposed to perform this culture mode in small-scale systems [6]. For instance, diffusive glucose release from a polymeric matrix has been applied in shake flasks [7, 8] and microtiter plates [9]. Another alternative consists on the enzymatic release of glucose from a polymeric substrate that cannot be degraded by *E. coli* [10, 11]. However, in such systems the specific growth rate cannot be controlled via glucose supply. Microfluidic devices have been implemented to perform fed-batch schemes in microtiter plates [12]. Other devices have been proposed to feed glucose solutions to shake flasks [13, 14]. The specific growth rate can be efficiently controlled in mini bioreactors using mathematical models coupled to feeding strategies [15]. Nevertheless, the latter systems are expensive and require additional equipment.

An alternative to the fed-batch mode to avoid overflow metabolism is the use of mutant strains with reduced glucose import capacity. Glucose is first transported from the extracellular medium to the periplasmic space of *E. coli* by porins and then internalized into the cytoplasm by the phosphoenolpyruvate-dependent phosphotransferase system (PTS) [16]. When the external glucose concentration is lower than 1 µM, or when the PTS is inactive, proteins normally involved in the transport of other carbohydrates such as the galactose: H^+^ symporter GalP and the high-affinity ABC transporter Mgl system are induced in *E. coli* [17]. Therefore, eliminating or reducing the activity of the PTS has been applied to decrease acetate formation. This strategy has been implemented by deleting genes encoding components of the PTS [18, 19] or by repressing their expression by means of non-coding mRNA [20]. We have previously reported that the inactivation of PTS and overexpression of the galactose transporter GalP efficiently reduced overflow metabolism and allows attaining high cell-densities in batch mode using up to 120 g/L glucose [21–25]. Fuentes and co-workers developed a library of mutant strains with single or combinatory deletions in genes encoding the PTS permeases and common components (*manX*, *malX*, *nagE*, *bglF*, *ptsG*, *ptsHIcrr*), as well as non-PTS transporters (*galP* and *mglABC*). Such mutations resulted in slower rates of glucose import and consequently, a strong reduction of acetate production and growth rate [26]. Compared to their wild type, some of these strains have the capacity to produce higher amounts of plasmid DNA [26] or recombinant protein [27]. However, their slow growth rate (from 21 to 72% lower than the wild type [26]) in mineral medium is a disadvantage for developing high cell-density cultures in batch mode. Therefore, modification of the culture media is key to increase the growth rate of the engineered strains while keeping their low aerobic acetate synthesis.

In the present work, we propose a simple strategy to mimic fed-batch mode using the strains library of Fuentes and co-workers when cultured in batch mode. Fast growth rate in these strains was promoted by adding extra nutrients to a chemically defined medium. Upon the exhaustion of key nutrients, the cells shifted to slow growth, similar to a fed-batch. The strains were characterized for constitutive GFP expression in microtiter plates using 10 or 20 g L^−1^ glucose. The best GFP producer strain was then cultured in 2 L bioreactors with 100 g L^−1^ glucose in batch mode. The wild type strain W3110 was cultured in an exponentially fed-batch culture at the same growth rate than the mutant strain WHIC using the same total amount of glucose. The mutant strain displayed the biphasic growth and produced only small amounts of acetate, while doubling the amount of GFP produced in fed-batch cultures when compared to the wild-type and reaching more than 50 g L^−1^ of biomass. Therefore, the library of mutants can be used to mimic fed-batch cultures in batch mode, which are useful to accelerate early bioprocess development.

## Results and discussion

### High throughput screening of *E. coli* strains with reduced glucose import capacity

The strains employed in this study were taken from the library of mutants developed by Fuentes and coworkers [25]. The genotype of the strains is shown in Table 1. All strains were transformed with plasmid pWF14, which carries a gene encoding *gfp* under transcriptional control of a constitutive promoter, as detailed in “Methods” section. Aerobic cultures were performed in mineral medium with yeast extract and glucose. Two different glucose concentrations were used: 10 and 20 g L^−1^, supplemented with 4 or 8 g L^−1^ of yeast extract, respectively. Cultures were performed in baffled microtiter plates where the oxygen transfer rate can be higher than 100 mmol L^−1^ h^−1^ [28].

**Table 1:** Genes inactivated in each of the strains used in this study

Strain	Inactivated genes
W3110	Wild type
WG	*ptsG*
WGX	*ptsG*, *malX*
WGB	*ptsG*, *bglF*
WGE	*ptsG*, *nagE*
WGM	*ptsG*, *manX*
WGMX	*ptsG*, *manX*, *malX*
WGMB	*ptsG*, *manX*, *bglF*
WGME	*ptsG*, *manX*, *nagE*
WGP	*ptsG*, *galP*
WGC	*ptsG*, *mglABC*
WGMP	*ptsG*, *manX*, *galP*
WGMC	*ptsG*, *manX*, *mglABC*
WHI	*ptsHIcrr*
WHIP	*ptsHIcrr*, *galP*
WHIC	*ptsHIcrr*, *mglABC*

**Fig. 1:**
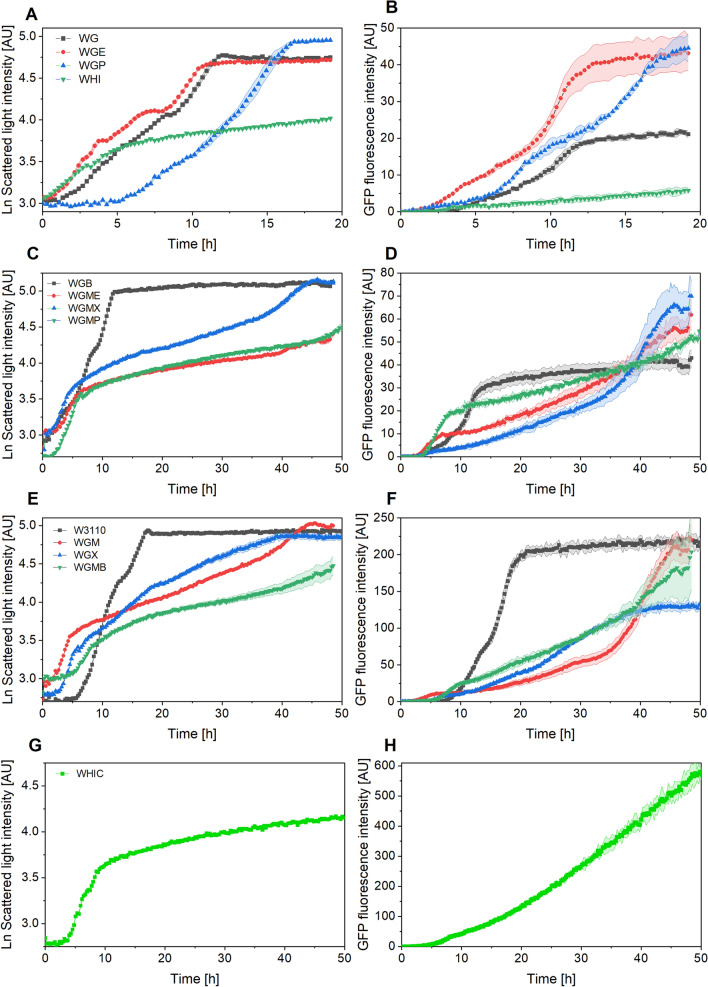
Cultures of *E. coli* strains constitutively expressing the GFP in semi-defined medium supplemented with 10 g L^−1^ glucose and 4 g L^−1^ yeast extract. The left panel shows scattered light intensities, and the right panel shows GFP fluorescence intensities. **A**, **B** Cultures of strains WG, WGE, WEP and WHI; **C**, **D** cultures of strains WGB, WGME, WGMX, and WGMP; **E**, **F** cultures of W3110, WGM, WGX and WGMB; **G**, **H** cultures of strain WHIC. Culture conditions: 48 well FlowerPlates®, *V*_*L*_ = 800 µL, *n* = 1500 rpm, *d*_0_ = 3 mm. Note that the scales are not identical. Shaded bands indicate the standard deviation between triplicate experiments

**Fig. 2:**
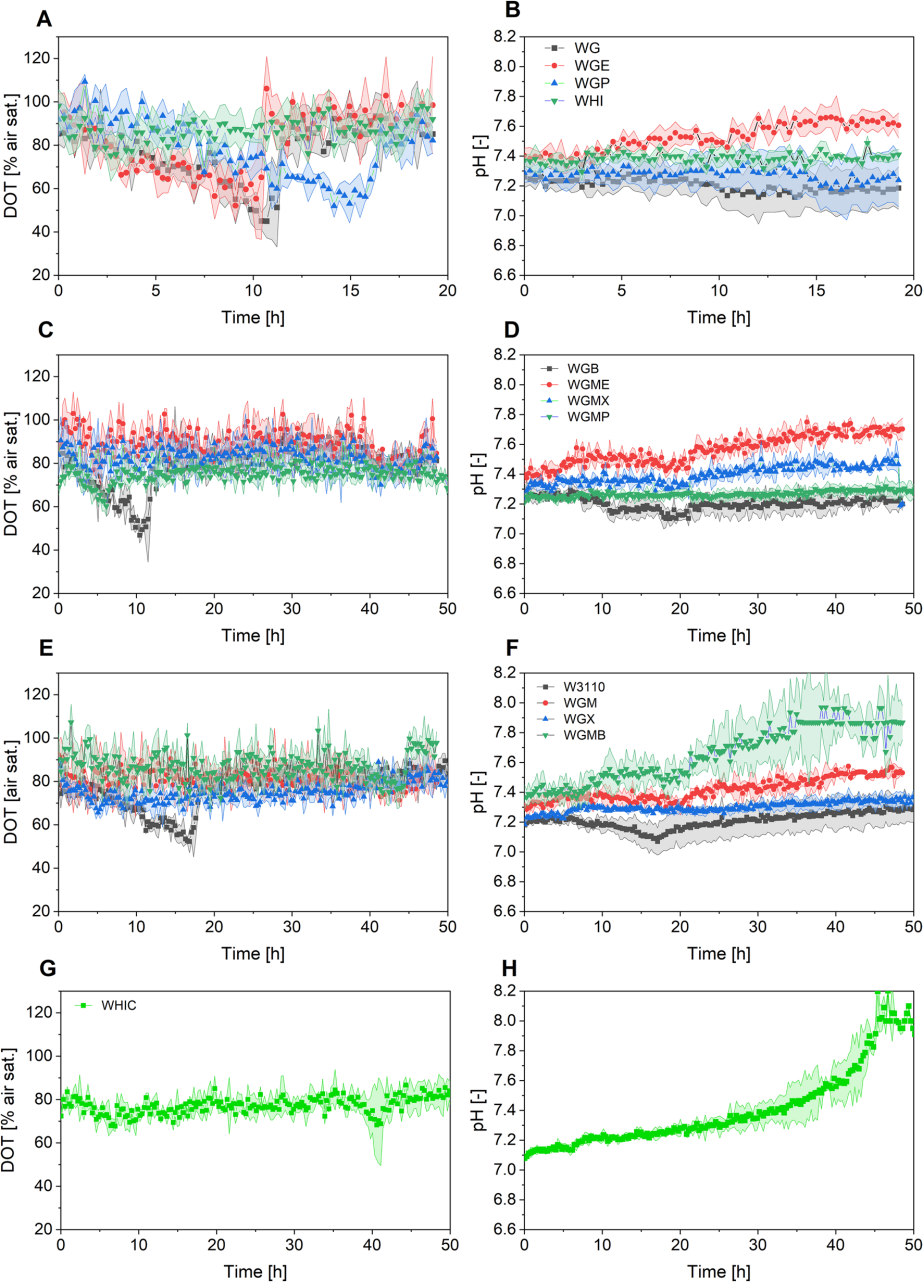
Cultures of *E. coli* strains constitutively expressing the GFP in semi-defined medium supplemented with 10 g L^−1^ glucose and 4 g L^−1^ yeast extract. The left panel shows the Dissolved oxygen tension (DOT), and the right panel shows pH values. **A**, **B** Cultures of strains WG, WGE, WEP and WHI; **C**, **D** cultures of strains WGB, WGME, WGMX, and WGMP; **E**, **F** cultures of W3110, WGM, WGX and WGMB; **G**, **H** cultures of strain WHIC. Culture conditions: 48 well FlowerPlates®, *V*_*L*_ = 800 µL, *n* = 1500 rpm, *d*_0_ = 3 mm

Figure 1 shows the biomass and GFP fluorescence profiles in cultures with 10 g L^−1^ glucose. The levels of GFP fluorescence exhibited important differences between strains. Therefore, the results are grouped depending on the GFP fluorescence obtained. The strains WGP, W3110, WGMX and WHIC presented *lag* phases ranging from 1 to 5 h (Fig. 1, left panel). All the strains had a fast growth rate phase, followed by a slow growth rate phase, which may occur due to depletion of glucose and yeast extract nutrients, as discussed below. The growth rates of both phases are reported in Table 2. The maximum scattered light intensity (ScL) reached ranged from 56 to 174 (Fig. 1). The mutant strains displayed GFP fluorescence lower than that of the wild type (Fig. 1, right panel), with the exception of WHIC, that reached a GFP fluorescence 2.7-fold greater than W3110. Although the extracellular glucose was not monitored, it was considered that the cultures had a very low metabolic activity after little DOT changes (Fig. 2) and relatively stable GFP fluorescence signal. At this point, cultures were finalized.

**Table 2:** Specific growth rates of the strains (*µ*) in batch cultures with 10 g L^−1^ glucose. Subscripts 1 and 2 refer to the first and second growth phase, respectively

Strain	*µ*_1_ [h^−1^]	*µ*_2_ [h^−1^]
W3110	0.67 ± 0.03	0.22 ± 0.01
WG	0.54 ± 0.03	0.34 ± 0.02
WGB	0.42 ± 0.03	0.30 ± 0.01
WGE	0.52 ± 0.06	0.30 ± 0.01
WGM	0.56 ± 0.05	0.05 ± 0.01
WGMB	0.61 ± 0.27	0.06 ± 0.01
WGME	0.38 ± 0.06	0.03 ± 0.00
WGMP	0.62 ± 0.03	0.04 ± 0.01
WGMX	0.47 ± 0.02	0.05 ± 0.00
WGP	0.71 ± 0.03	0.35 ± 0.05
WGX	0.64 ± 0.02	0.07 ± 0.01
WHI	0.53 ± 0.07	0.11 ± 0.01
WHIC	0.46 ± 0.01	0.04 ± 0.01

The microtiter plates included optodes for dissolved oxygen (DOT) and pH monitoring online. As shown in Fig. 2, DOT remained above 20%, and between 6.9 and 7.9 in all cultures. Therefore, it can be assumed that the cultures were not oxygen limited and that pH was not a stress factor.

**Fig. 3:**
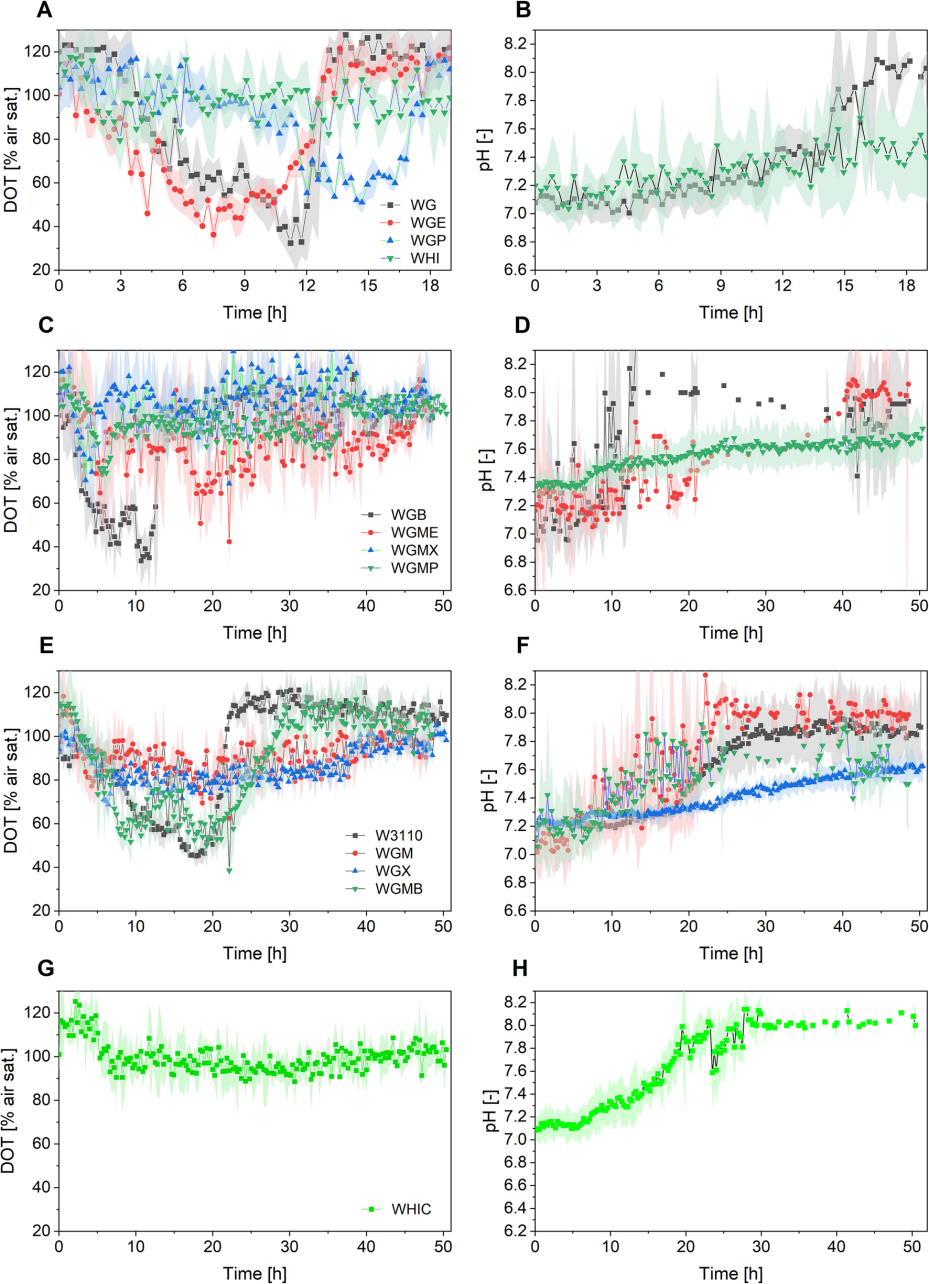
Cultures of *E. coli* strains constitutively expressing the GFP in semi-defined medium supplemented with 20 g L^−1^ glucose and 8 g L^−1^ yeast extract. The left panel shows scattered light intensities, and the right panel shows GFP fluorescence intensities. **A**, **B** Cultures of strains WG, WGE, WEP and WHI; **C**, **D** cultures of strains WGB, WGME, WGMX, and WGMP; **E**, **F** cultures of W3110, WGM, WGX and WGMB; **G**, **H** cultures of strain WHIC. Culture conditions: 48 well FlowerPlates®, *V*_*L*_ = 800 µL, *n* = 1500 rpm, *d*_0_ = 3 mm. Note that the scales are not identical. Shaded bands indicate the standard deviation between triplicate experiments

**Fig. 4:**
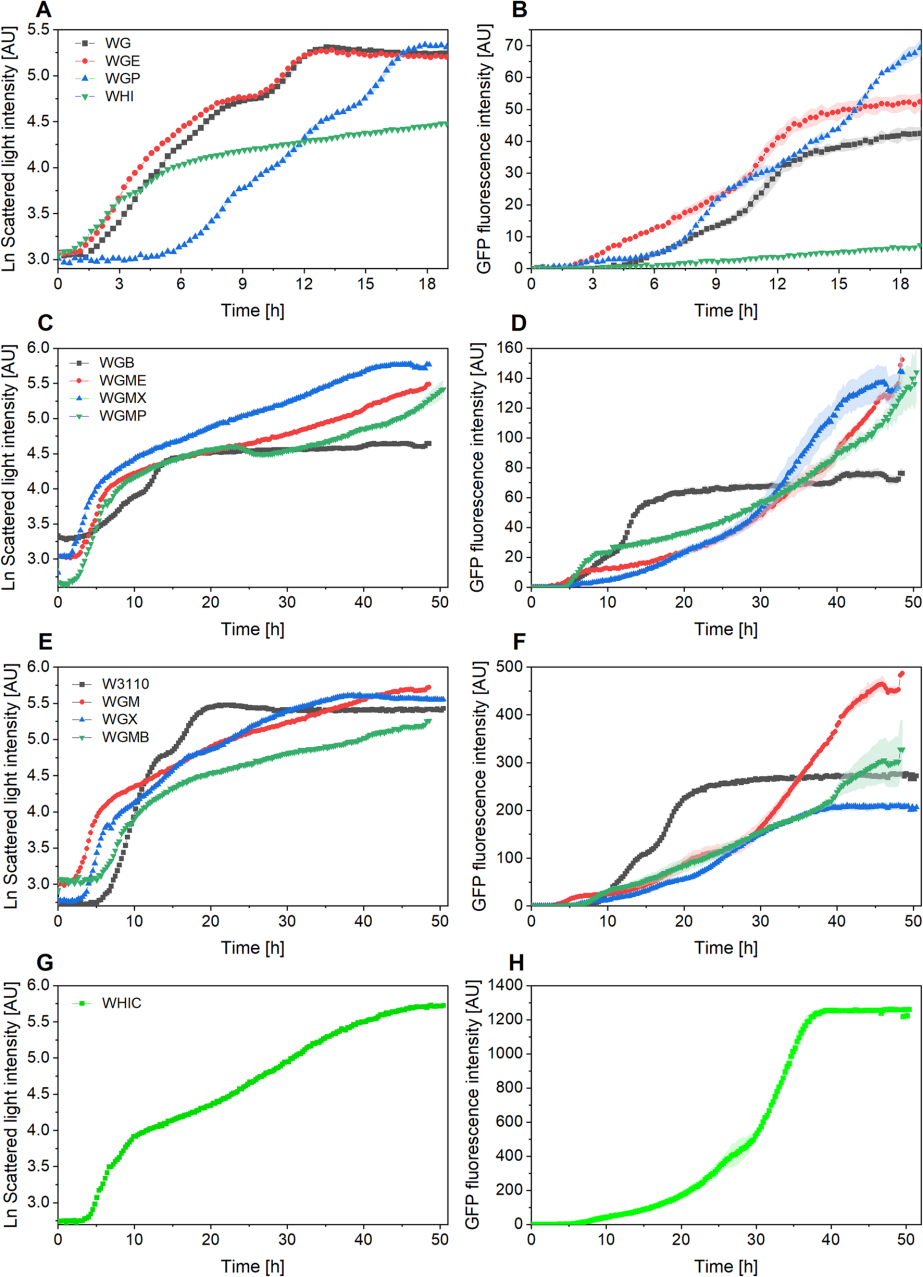
Cultures of *E. coli* strains constitutively expressing the GFP in semi-defined medium supplemented with 20 g L^−1^ glucose and 8 g L^−1^ yeast extract. The left panel shows the dissolved oxygen tension (DOT), and the right panel shows pH values. **A**, **B** Cultures of strains WG, WGE, WEP and WHI; **C**, **D** cultures of strains WGB, WGME, WGMX, and WGMP; **E**, **F** cultures of W3110, WGM, WGX and WGMB; **G**, **H** cultures of strain WHIC. Culture conditions: 48 well FlowerPlates®, *V*_*L*_ = 800 µL, *n* = 1500 rpm, *d*_0_ = 3 mm. pH data from WGE and WGP are not included due to failure of the readings

The strains were also evaluated using 20 g L^−1^ glucose with the aim of increasing the amount of biomass achievable in batch mode. The culture profiles are shown in Fig. 3, grouped as in Fig. 1, while DOT and pH profiles are shown in Fig. 4. Similar to cultures with 10 g L^−1^ glucose, the cells displayed two growth phases. The maximum ScL reached ranged from 89 to 323, nearly doubling the ScL signal of the previous cultures for each strain. Contrary to what was observed in cultures with a lower amount of glucose, three mutant strains reached GFP fluorescence values higher than that of the wild type. Namely, the GFP fluorescence in cultures of strains WGMB and WGM were 19 and 74% higher than that of W3110 (Fig. 3F). The better performance of these strains, which was not observed in cultures using 10 g L^−1^ glucose, could be attributed to their very low acetate production [26], which is more advantageous when a higher amount of glucose is used. Notably, the GFP fluoresce in cultures of strain WHIC was 4.5-fold greater when compared to W3110 (Fig. 3H). Fragoso et al. [27], reported cultures of strains WG, WGM, WGX, WGMC, WHI and WHIC in M9 medium expressing GFP. They found that all the mutant strains produced more GFP than W3110 in M9 medium supplemented with 20 g L^−1^ glucose, being WGM the best producer. In addition to the differences in medium composition, there are other factors possibly causing these contrasting results. Fragoso and coworkers [27] employed an IPTG to express GFP. The use of IPTG combined with the altered cAMP intracellular amount in the mutant strains, as result of the lower glucose uptake rate, could enhance the activation of the *lac* promoter [27].

**Fig. 5:**
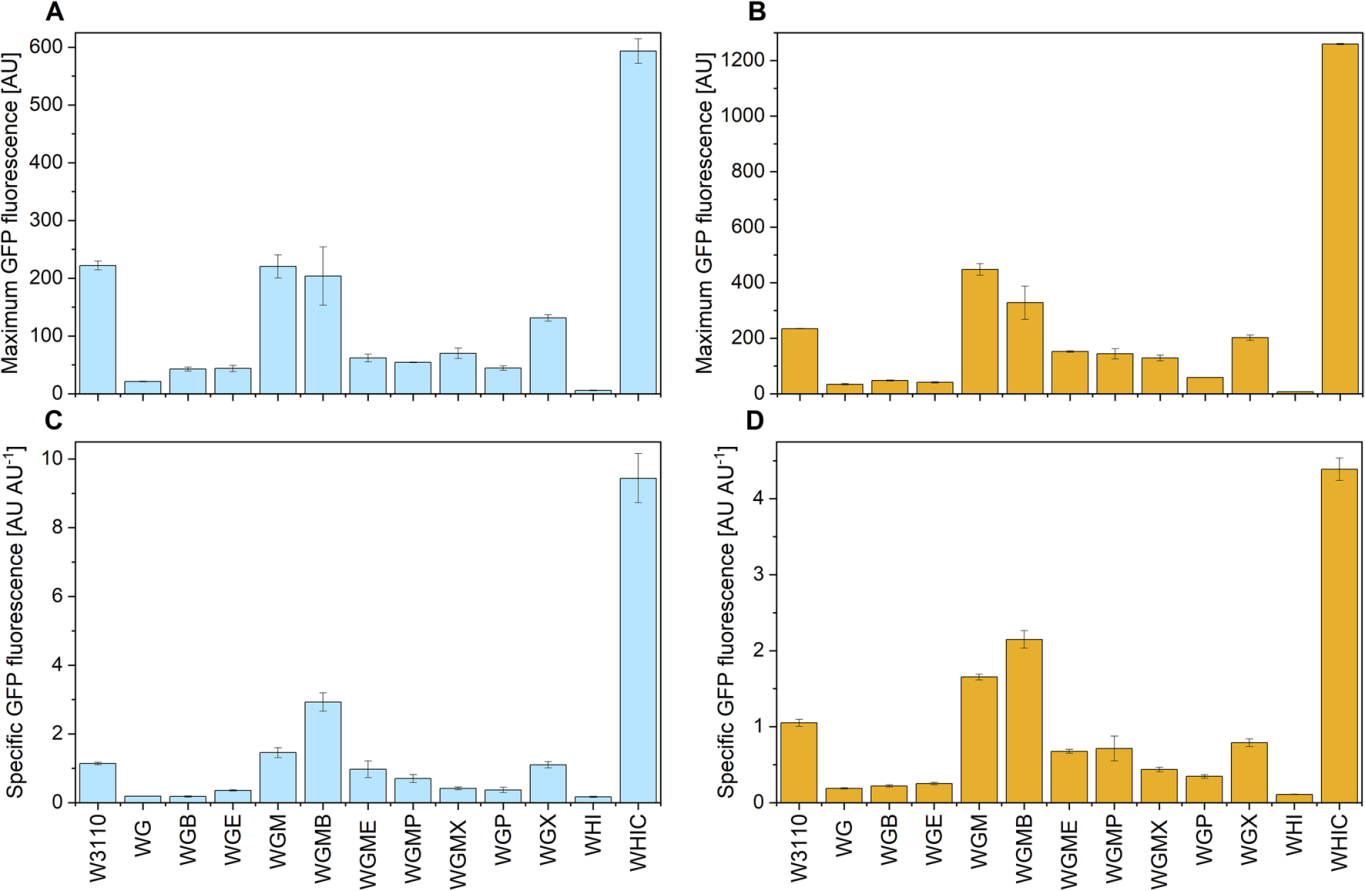
GFP expression performance of the wild type and mutant strains cultures in semi-defined medium supplemented with 10 g/L glucose and 4 g L^−1^ yeast extract (**A**, **B**) or 20 g L^−1^ glucose and 8 g L^−1^ yeast extract (**C**, **D**). Top panel: maximum GFP fluorescence; bottom panel: specific GFP fluorescence. Values were calculated over the slow-growth phase. Culture conditions: 48 well FlowerPlates. V_L_ = 800 µL, *n* = 1500 rpm, *d*_0_ = 3 mm. Note that the scales are not identical. Vertical lines indicate the standard deviation between triplicate experiments

This bias may be dependent on the specific strain, due to their particular characteristic of the glucose transport. Such effects are not expected in the constitutive system used here, although the strength of the used promoter has not been compared with that of the inducible promoter used by Fragoso and coworkers [27]. Figure 5 contains the maximum GFP fluorescence and the specific GFP fluorescence (SGF) reached in all cultures. Even though strains WGM and WGMB did not reach a GFP fluorescence level higher than that of W3110 in cultures with 10 g L^−1^ glucose, their SGF was greater than that of the wild type (Fig. 5A, C). Therefore, when higher cell densities are attained (presumably due to low acetate production), the GFP fluorescence in cultures of WGM and WGMB was higher than for W3110 (Fig. 5B, D). Interestingly, strain WHIC displayed considerably higher GFP fluorescence and SGF than all the other strains, but its SGF decreased when the glucose concentration was increased from 10 to 20 g L^−1^ (Fig. 5B, D).

Samples were taken at the end of the cultures with 20 g L^−1^ glucose to measure the amount of GFP produced. Table 3 shows the specific growth rates and GFP fluorescence during the two growth phases, and the final GFP concentration in each culture. With the exception of WGB, WGE and WGP, the mutant strains reached specific growth rates similar to those of W3110 during the first growth phase (*µ*_1_).

**Table 3:** Specific growth rates and final GFP concentration in batch cultures with 20 g L^−1^ glucose. ND: Not detected by the method used; *µ*: specific growth rate; SGF: specific GFP fluorescence. Subscripts 1 and 2 refer to the first and second growth phase, respectively

Strain	*µ*_1_ [h^−1^]	SGF_1_ [–]	*µ*_2_ [h^−1^]	SGF_2_ [–]	Final GFP conc. [mg L^−1^]
W3110	0.70 ± 0.03	1.00 ± 0.08	0.12 ± 0.01	1.24 ± 0.09	1.5 ± 0.0
WG	0.52 ± 0.03	0.20 ± 0.01	0.24 ± 0.02	0.24 ± 0.01	ND
WGB	0.43 ± 0.03	0.20 ± 0.00	0.26 ± 0.01	0.29 ± 0.01	ND
WGE	0.42 ± 0.02	0.21 ± 0.01	0.23 ± 0.01	0.31 ± 0.01	ND
WGM	0.66 ± 0.04	0.53 ± 0.02	0.04 ± 0.00	1.28 ± 0.08	1.91 ± 0.40
WGMB	0.61 ± 0.27	1.00 ± 0.05	0.03 ± 0.00	2.73 ± 0.23	1.35 ± 0.80
WGME	0.59 ± 0.06	0.28 ± 0.01	0.04 ± 0.00	0.90 ± 0.01	0.82 ± 0.21
WGMP	0.46 ± 0.03	0.66 ± 0.03	0.03 ± 0.01	0.95 ± 0.10	0.53 ± 0.04
WGMX	0.66 ± 0.02	0.13 ± 0.01	0.05 ± 0.00	0.63 ± 0.05	0.48 ± 0.04
WGP	0.43 ± 0.03	0.77 ± 0.06	0.31 ± 0.01	0.35 ± 0.01	ND
WGX	0.94 ± 0.02	0.57 ± 0.05	0.07 ± 0.01	1.07 ± 0.09	0.82 ± 0.04
WHI	0.51 ± 0.07	0.05 ± 0.01	0.04 ± 0.00	0.22 ± 0.01	ND
WHIC	0.46 ± 0.01	1.73 ± 0.01	0.06 ± 0.00	6.05 ± 0.02	5.15 ± 0.76

**Fig. 6:**
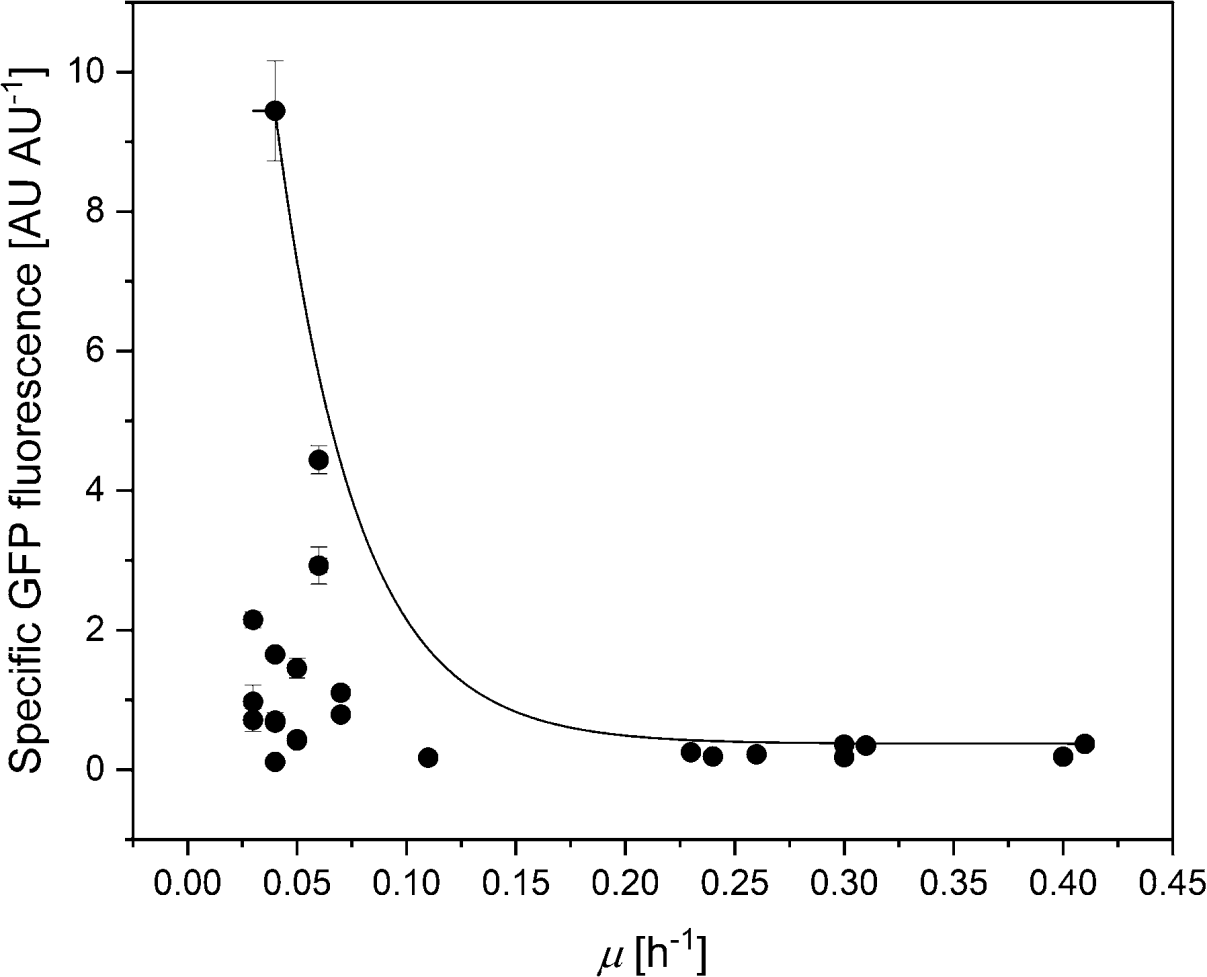
Specific GFP fluorescence vs. specific growth rate (*µ*) of the different mutant strains and culture conditions in microbioreactors. Vertical lines indicate the standard deviation between triplicate experiments

The growth rate during the second growth phase (*µ*_2_) was considerably lower for the mutant strains, compared to *µ*_1_. Namely, *µ*_2_ was reduced around 90% with respect to *µ*_1_ in strains WGM, WGMB, WGME, WGMX, WGMP, WGX. While these strains are derivatives of WG (Δ*ptsG*), the growth rate of WG during the second growth phase was less affected than in the double or triple mutants. The *manX* gene has been deleted in strains WGM, WGMB, WGME, WGMX and WGMP. Such gene codes for the mannose transporter and it can also import glucose [27]. The observed decrease in growth rate could be attributed to this deletion, since strains with deletions of genes *bglF* and *nagE* but with intact *manX* (WGB and WGE, respectively) the growth rate decrease was around 4% (Table 2). In strains WHI and WHIC, the *ptsHIcrr* operon has been inactivated. These strains also exhibited a reduction in growth rate of more than 9%, when compared to the first growth phase (Table 3). With the exception of WGP, the SGF of the second growth phase increased, compared to the first phase (Table 3). In general, this is similar to what is observed in fed-batch cultures, where a decrease in growth rate due to glucose limitation leads to increased recombinant protein yields [4, 5]. The amount of GFP quantified in the cultures agreed with the GFP fluorescence, but GFP was not detected by the method used in the case of the lowest GFP fluorescence (Table 3). Figure 6 shows the relationship between growth rate and SGF in all cultures. As can be seen, there seems to be a maximum SGF at a low growth rate, but then it decreases at the lowest growth rates. This agrees with previous studies, which suggest that the optimum growth rate depends on the specific produced protein [29, 30].

**Fig. 7:**
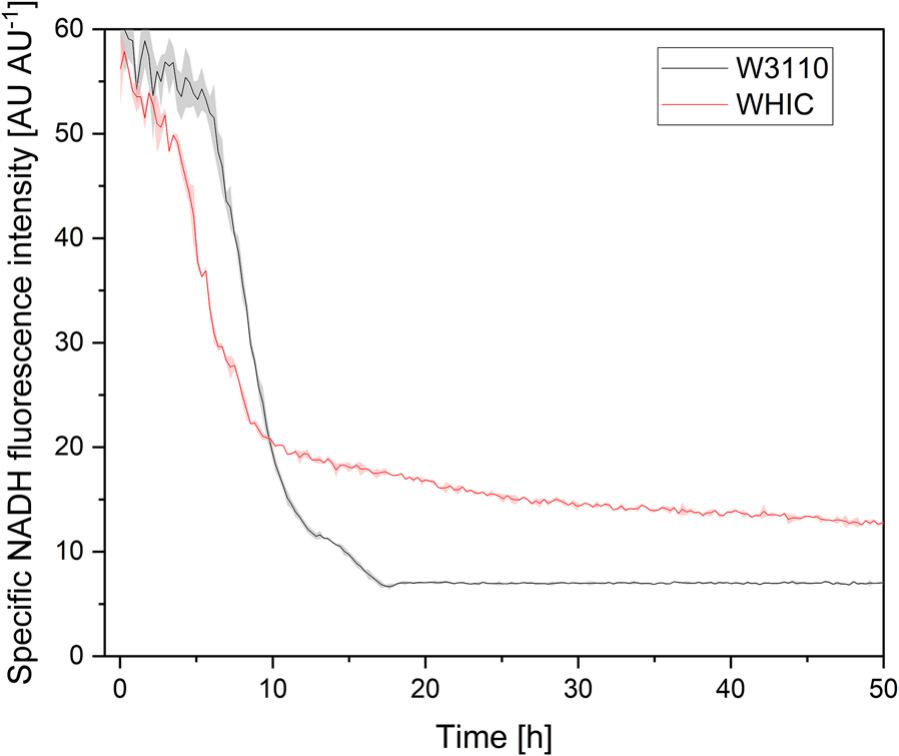
Specific NADH fluorescence of strains W3110 and WHIC in cultures in semi-defined medium supplemented with 10 g L^−1^ glucose and 4 g L^−1^ yeast extract. Culture conditions: 48 well FlowerPlates, *V*_*L*_ = 800 µL, *n* = 1500 rpm, *d*_0_ = 3 mm. Shaded bands indicate the standard deviation between triplicate experiments

The modification of glucose transport can also have metabolic consequences beyond reduction of growth rate. For instance, Fig. 7 shows the specific NADH fluorescence in cultures of W3110 and WHIC with 10 g L^−1^ glucose. The specific NADH fluorescence was similar for both strains at the beginning of the cultures, decreasing as time progressed, but at a lower rate for WHIC than for W3110. The NADH specific fluorescence decreased more slowly after a time that is coincident with the shift to a low growth rate (Fig. 3). It is possible that at this point, the energy demand to synthesize the depleted nutrients could increase the respiratory activity and thus the NADH consumption. Interestingly, the NADH specific fluorescence remained higher for WHIC than for W3110 since 10 h of culture (Fig. 7). It may occur due to the higher acetate production of strain W3110, since acetate is synthesized to supply NAD^+^ to glycolysis [1]. Moreover, during the acetate excretion, the pH gradient of the cell is uncoupled [1], and thus extra energy will be required to maintain the homeostasis. Furthermore, during the second growth phase, the respiratory activity of WHIC is expected to be lower than that of W3110 [18]. Taken together, these factors may explain the higher NADH fluorescence.

Based on the discussed results, strain WHIC was selected for high cell-density cultures in batch mode.

## WHIC batch cultures vs. W3110 fed-batch cultures

**Fig. 8:**
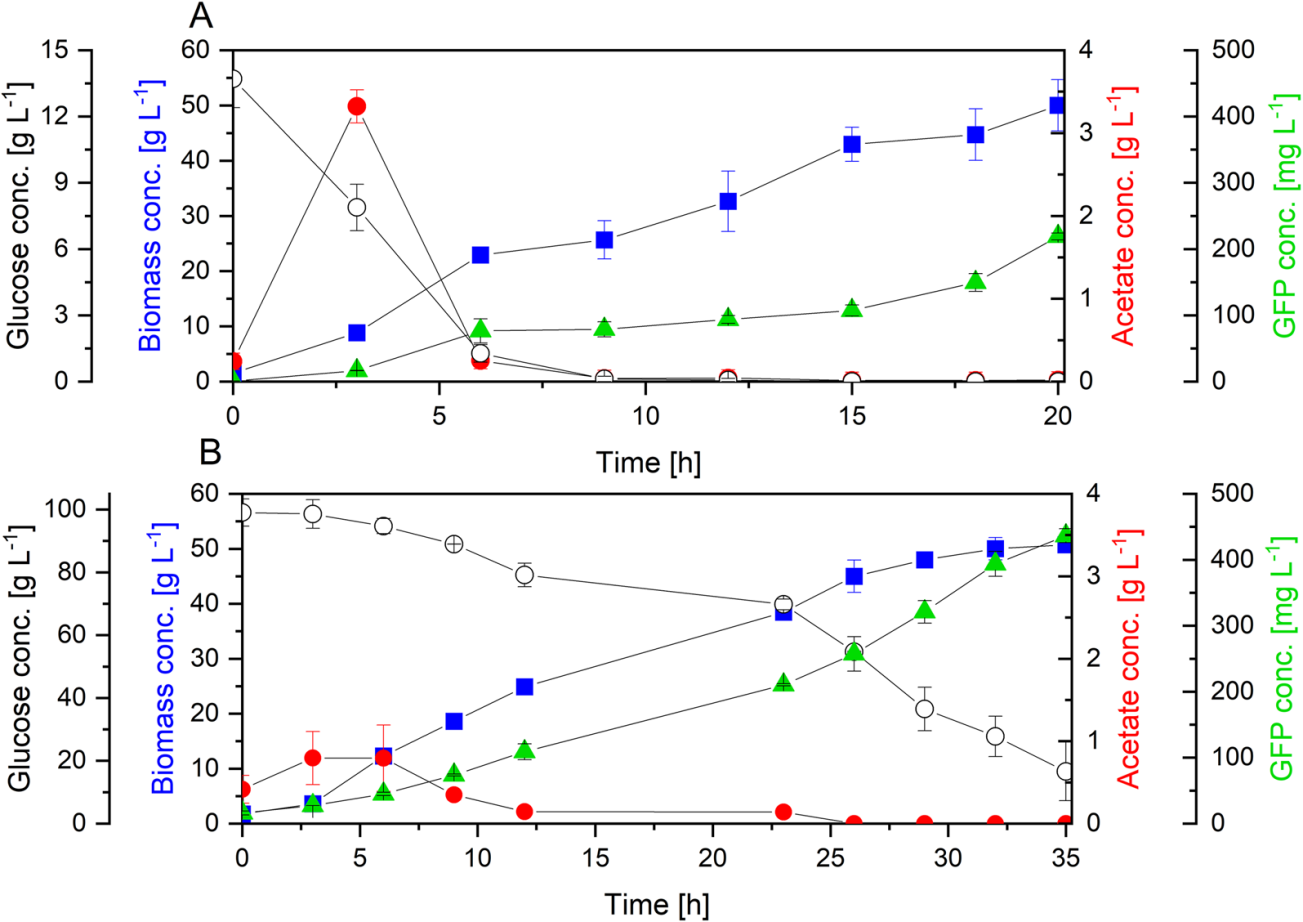
High cell-density cultures of W3110 (**A**) and WHIC (**B**). Strain W3110 was cultured in fed-batch mode with an exponentially increasing feeding rate of 0.05 h^−1^, and WHIC in batch mode. The total amount of glucose used in both cultures was 100 g L^−1^. Empty circles: glucose; filled circles: acetate; squares: biomass; triangles: GFP concentrations. Vertical lines show the experimental error in duplicate cultures

To further assess the potential of the WHIC strain for high cell-density cultures with reduced acetate accumulation and improved recombinant protein production, batch cultures were performed and compared to W3110 fed-batch cultures. To better compare the performance of these strains, W3110 was cultured in exponentially fed-batch mode the attain an specific growth rate of 0.05 h^−1^, to match the growth rate of WHIC during the second growth phase. Strain WHIC was cultured using 100 g L^−1^ glucose. The amount of yeast extract and total amount of glucose were the same for both cultures. The culture profiles are shown in Fig. 8. Cultures of W3110 showed the typical profile of a fed-batch culture (Fig. 8A). W3110 grew at *µ* of 0.46 ± 0.02 h^−1^ during the batch phase, and acetate was accumulated. This phase of acetate production occurred during the first 3 h of culture (Fig. 8A), which was consumed during the first hours of the glucose-limited phase (acetate consuming phase, from 3 to 8 h). Acetate was the only by-product detected for both strains. The acetate concentration remained below detectable levels after this and no glucose accumulation was observed, as expected for an exponentially fed-batch scheme. Mutant WHIC showed again two growth phases (Fig. 8B). The first phase lasted 6 h with a *µ* of 0.33 ± 0.04 h^−1^, while the second phase lasted 26 h with a *µ* of 0.04 ± 0.01 h^−1^. The growth rate during the first phase was approx. 30% lower for both strains, compared to cultures using 20 g L^−1^ glucose (Table 3), which could be attributed to the osmotic stress caused by the concentrated medium [24]. At the point of growth rate decrease, the biomass concentration of WHIC was 14.4 g L^−1^. Based on the typical protein content in *E. coli* [31], and the amino acid content in the proteins of *E. coli* [32], we estimated the amount of *E. coli* biomass that could be synthesized exclusively using the amino acids from the yeast extract. Based on the yeast extract composition reported by Popdora and coworkers [33], it was estimated that proline and glycine could limit the formation of biomass, since their availability could sustain the synthesis of only 10.4 and 7.52 g L^−1^ of biomass, respectively. Despite the variations in composition of the yeast extract and exact amino acids composition of strain WHIC, this rough estimation illustrates that the assumption that growth rate decrease occurs due to amino acids depletion is reasonable.

Bäcklund et al. [18] also compared the performance of W3110 and a PTS mutant for recombinant protein production. They showed that the specific production rate of beta-galactosidase was similar for W3110 in fed-batch and the mutant strain in batch cultures. However, the growth profiles were very different in both cultures, and cell densities were approx. 10 g L^−1^ for W3110 and less than 5 g L^−1^ for the mutant strain. In contrast, as shown in Fig. 8, no important differences were observed in the final biomass concentration between W3110 and WHIC, reaching 48 and 53.5 g L^−1^ of biomass, respectively in our experiments. Remarkably, there were significant differences in the amounts of acetate accumulation, GFP titer, and *Y*_*GFP/X*_. Acetate concentration in cultures of W3110 reached a maximum of 3.3 g L^−1^, whereas acetate production was 64% lower for WHIC strain, reaching only 1.2 g L^−1^. GFP final concentration was twofold higher in WHIC than in W3110, while *Y*_*GFP/X*_ and specific rate of GFP production (*q*_*GFP*_) were 77 and 14.5% higher in WHIC compared to W3110 during the phase of no acetate production (8–20 h for W3110 and 23–35 h for WHIC). The fact that more GFP is produced by WHIC, despite the fact that acetate was not produced by any strain in this phase, can be explained by the metabolic differences of the mutant strain. When glucose is transported by the PTS (like in W3110), one ATP mol is consumed per mol of glucose that is internalized, and glucose 6-phosphate is formed during the transport of glucose inside the cell. In this case, the phosphate group is transferred from phosphoenolpyruvate. In the mutant strain, glucose is phosphorylated by the glucokinase. Thus more PEP is available for energy generation and biosynthesis. Such differences have in general, positive effects from a bioprocessing perspective [17], which can explain the results from Fig. 8. The overall volumetric GFP productivity was 11.0 ± 1.0 mg L^−1^ h^−1^ in cultures of W3110, while reached 12.9 ± 1.1 mg L^−1^ h^−1^ in cultures of WHIC. Therefore, despite the longer cultivation time required, the engineered strain performed better than its wild type.

## Conclusion

The presented results demonstrate that, in general, a typical fed-batch performance can be mimicked using engineered strains cultured in batch mode. This is valid at least for the specific expression system and protein produced, but in principle could be extended to other recombinant proteins, where the initial screening could result in a different optimum growth rate/specific mutant for culture scale-up. Moreover, it was demonstrated that batch cultures of the engineered strain with high glucose concentration can perform better than fed-batch cultures of the wild type strain at the same growth rate. Batch operations are easier to perform than fed-batch, which make attractive the use of the mutant strains presented. Furthermore, a glucose-feeding pump will not be necessary for cultures of the mutant strains, which would reduce the energy consumption during the process. Therefore, we consider that this approach is a valuable alternative for accelerated high-throughput screening and culture scale-up.

## Methods

### Plasmid

Plasmid pWF14 (3824 bp) expresses the supergloGFP (sgGFP) green fluorescent protein from a constitutive promoter. This plasmid contains genes conferring resistance to kanamycin and chloramphenicol and the pUC origin of replication. Protein sgGFP contains four amino acid changes (F64L, S65C, I167T y K238N) when compared to the sequence from the GFP from *Aequorea victoria* (avGFP) [34]. At 37 °C, sgGFP displays better folding and fluorescence when compared to avGFP. The synthetic constitutive promoter in pWF14 was generated from a design based on a consensus sequenced derived from 46 promoters [34]. The 42 bp promoter sequence was further modified by random insertion mutagenesis of each of the four DNA bases dA, dC, dG or dT. The promoter library was generated by conventional oligonucleotide chemical synthesis. The random insertional mutagenesis was performed by employing a diluted equimolar mixture of the four bases and a synthesis cycle lacking the capping step. A library was generated by ligating the mutant promoters to a plasmid vector. Selection of clones from the random library was based on detecting the transformed bacterial colonies with the highest fluorescent level. Plasmid pWF14 was selected as it displayed the highest fluorescence level in the library. The sequencing of the promoter region in pWF14 showed the insertion of an A (adenine) in the spacer region. The complete sequence and features of pWF14 is provided in Additional file 1.

## Bacterial strains and culture media

The *E. coli* strains used in this work are described in Table 1. The wild type strain W3110 is a derivative of K-12. All the mutants are derivatives of W3110. Strains were transformed with pWF14 and preserved in 40% glycerol stocks at − 70 °C. All cultures were performed in a semi defined mineral medium containing glucose and yeast extract. The composition of the mineral medium (in g L^−1^) was: K_2_HPO_4_, 17; KH_2_PO_4_, 5.3; (NH_4_)_2_SO_4_, 2.5; NH_4_Cl, 1.0; Citrate-Na_3_·2H_2_O, 2; MgSO_4_·7H_2_O, 1.0; Thiamine-HCl, 0.01. The medium was supplemented with 2 mL L^−1^ trace element solution and either 0.05 g L^−1^ kanamycin sulfate or 0.03 g L^−1^ chloramphenicol. The trace element solution composition (in g L^−1^) was: ZnCl_2_, 10.5; Na·EDTA, 5.5; CoSO_4_·7H_2_O, 1.5; MnSO_4_·H_2_O, 6.4; CuSO_4_·5H_2_O, 1.1; H_3_BO_3_, 1.5; Na_2_MoO_4_·2H_2_O, 1; FeCl_3_·6H_2_O, 51.4; and Fe(III)Cit-H·H_2_O, 39.9. Glucose, trace elements solutions and salt solutions were sterilized separately. Kanamycin, chloramphenicol, and thiamine solutions were sterilized by filtration and kept frozen at − 20 °C. The pH of the medium was adjusted to 7.4 before sterilization. The strains were precultured overnight in shake flasks with mineral medium containing 5 g L^−1^ glucose and 2 g L^−1^ yeast extract. Aliquots were taken to inoculate the main cultures at an initial optical density (OD_600nm_) of approx. 0.1. All the chemicals were purchased to Sigma Aldrich (MO, USA).

## Microbioreactor cultures

Microtiter plates cultures were performed in a Multiparameter FlowerPlate (m2p-labs GmBH, Bäsweiler, Germany) using the semi defined medium containing 10 or 20 g L^−1^ glucose, plus 0.4 g of yeast extract per g of glucose. The microtiter plates were incubated at 37 °C in a BioLector (m2p-labs GmBH, Bäsweiler, Germany) with filling volume of 800 µL and a constant orbital shaking of 1500 rpm, shaking diameter 3 mm. Biomass was measured by scattered light (ScL) at 620 nm, using a gain of 15. NADH fluorescence was measured with excitation and emission wavelengths of 365 and 450 nm, respectively with a gain of 90. GFP production was followed at 488 nm for excitation and 520 nm for emission and a gain of 85. Specific GFP fluorescence intensity (SGF) was calculated as the slope of the curve of ScL intensity vs. GFP fluorescence intensity over the time involved. All cultures were carried out in triplicate. The total volume of each well was taken at the end of the cultures and stored at − 20 ° C until further analysis.

## 2 L stirred tank bioreactor cultures

Fed-batch cultures were carried out in a 2 L Biostat B Plus stirred tank bioreactor (Sartorius, Göttingen, Germany) equipped with controls for pH, temperature, agitation, and dissolved oxygen. NH_4_OH and H_2_PO_4_ solutions were automatically added to control pH at 7.0. Temperature was maintained at 37 °C, DOT was maintained above 30% by increasing the stirrer speed and airflow. The batch phase started with 15 g L^−1^ glucose and 50 g L^−1^ yeast extract in 0.5 L mineral medium supplemented with 0.05 g L^−1^ kanamycin sulfate. The fed-batch phase was started after 9 h, when initial glucose depletion was evidenced by a sudden increase of the DOT and pH signals. A concentrated 500 g L^−1^ glucose solution supplemented with 0.05 g L^−1^ kanamycin was added to the medium using a Watson–Marlow 101U/R programmable peristaltic pump. The feeding rate was programmed to maintain a specific growth rate of 0.05 h^−1^ using the following equation:

1 $$\text{F}=\left(\frac{{\upmu}_{\text{set}}}{{Y}_{x/s}}+{m}_{s}\right)\frac{xV{e}^{{\mu }_{set\left(t\right)}}}{{S}_{i}}$$

where *F* is the glucose feeding rate (L h^−1^), *Y*_*x/s*_ is the biomass yield on glucose, *m*_*s*_ is the specific consumption rate of glucose associated with maintenance (0.059 g g^−1^ h^−1^), *V* is the actual volume (L), *x* is the biomass concentration (g L^−1^), *µ*_*set*_ is the desired specific growth rate, *t* is the feeding elapsed time (h) and *S*_*i*_ is the glucose concentration in the feeding solution. Biomass concentration was determined as the dry cell weight. Samples were taken periodically for offline analyses. Fed-batch cultures were performed in duplicate.

High cell-density cultures of strain WHIC in batch mode were performed a 2 L Biostat B Plus stirred tank bioreactor (Sartorius, Göttingen, Germany) with 0.6 L of mineral medium containing 100 g L^−1^ glucose, 50 g L^−1^ yeast extract and 0.05 g L^−1^ kanamycin. DOT and pH were controlled as described above. Biomass concentration was determined as the dry cell weight. Samples were taken periodically for offline analyses. Batch cultures were performed in triplicate. For the stirred tank bioreactor cultures, GFP production was monitored by fluorescence readings in a ChronosBH Fluorometer (ISS Inc., Champaing, EUA). Wavelengths of 480 and 510 nm were used for excitation and emission, respectively. GFP was quantified as explained below.

## Metabolite analysis

Glucose and acetate were quantified by a chromatographic method in a Varian ProStar 210 (Varian Inc., California, EUA) HPLC system equipped with an Aminex HPX-87 H column (Bio-Rad Laboratories, California, EUA). The separation was carried out at 0.5 mL min^−1^ at 60 °C, and metabolites were detected in a spectrophotometric detector UV-Vis Varian PS-235 (Varian Inc., California, EUA) at 210 nm.

## GFP quantification

Cells recovered from the cultures were used to quantify GFP production. For cell lysis, a 10 g L^−1^ lysozyme solution was added to the sample and then incubated at 37 °C for 15 min. Later, the cells were frozen at − 70 ° C for 30 min and thawed at room temperature. After centrifugation at 13,000 rpm for 2 min, the supernatant was recovered. GFP quantification was performed by microfluidics electrophoresis using the commercial Agilent Protein 80 Kit package (Agilent Technologies, California, USA) according to the manufacturer’s recommendations. For both quantifications, a standard curve of GFP concentration (rGFP, Sigma-Aldrich, MO, USA) was used against the area under the curve provided by the Agilent Expert software (Agilent Technologies, California, USA) or the emitted fluorescence units.

## Supplementary Information

**Additional file 1.** Plasmid pWF14 sequence.

## Data Availability

All data generated or analysed during this study are included in this published article and its Additional file 1.
